# The Effect of Lateralization of a Pelvic Brim Plate on the Fixation of an Anterior Column Fracture: A Biomechanical Analysis

**DOI:** 10.7759/cureus.24158

**Published:** 2022-04-15

**Authors:** Mehmet Burak Gökgöz, Bahadır Alemdaroğlu, Ahmet Özmeriç, Serkan İltar, Fatma K Erbay, Teyfik Demir

**Affiliations:** 1 Orthopedics and Traumatology, Sivas Numune Hospital, Sivas, TUR; 2 Orthopedics and Traumatology Department, Sağlık Bilimleri Üniversitesi (SBU) Ankara Training and Research Hospital, Ankara, TUR; 3 Department of Biomedical Engineering, Union of Chambers and Commodity Exchanges of Turkey (TOBB) University of Economics and Technology, Ankara, TUR; 4 Department of Mechanical Engineering, Union of Chambers and Commodity Exchanges of Turkey (TOBB) University of Economics and Technology, Ankara, TUR

**Keywords:** open reduction internal fixation, biomechanical, suprapectineal plate, anterior column, acetabular fractures

## Abstract

Objectives

Anterior column fractures can be seen as either isolated or accompanied by many types of complex acetabulum fractures. The aim of this study was to biomechanically compare the stability of a standard pelvic brim plate with a more laterally located suprapectineal plate, which is more commonly used in minimally invasive application, on an intermediate height anterior column fracture model under dynamic and static loading.

Materials and methods

Right side, adult, foam cortical shell artificial hemipelvis models were used (Sawbones, Pacific Research Laboratories, Vashon, WA, USA). Twenty-four (24) pieces of foam cortical shell artificial hemipelvis models were separated into three groups (M, L, and control). In group “M,” a suprapectineal plate was placed medially just adjacent to the pelvic brim. In group “L,” a laterally located suprapectineal plate was placed 2 cm lateral of the pelvic brim at its most proximal point. Then, dynamic load testing of 1000 cycles between 50 N and 500 N force and a static load test of 1.2 kN at 2 mm/minute were applied. Dynamic and static tests were conducted on an axial compression device. Displacements were measured after dynamic and static loading conditions.

Results

In the dynamic loading test at the AL point (superior intersection of the fracture line with the acetabular roof), the median displacement was significantly higher in group L than in group M (0.12 (IQR: 0.058-0.8125) mm and 0.04 (IQR: 0.03-0.065) mm (p = 0.02)). There was no other statistically significant difference in the displacement amounts in both dynamic and static loading conditions at other measurement points. The comparison of the stiffness of the M and L groups showed no statistically significant results, while the control group was significantly more rigid than both the M and L groups (p = 0.04 for both). None of the artificial hemipelvis models was found to be fractured at the end of the test.

Conclusion

Suprapectineal plates, placed on either the medial or lateral aspect of the pelvic brim, may be used for the fixation of anterior column-type fractures to provide rigid fixation and stability. As plate location did not impact stiffness and stability, the results suggest that surgeons have flexibility in determining the fixation based on accessibility, fracture pattern, and surgeon experience.

## Introduction

Surgical treatment of displaced acetabular fractures has become widespread over the last 30 years, following the studies of Letournel on the diagnosis, classification, and treatment approach [1]. Since the studies by Letournel, the current standard treatment of acetabular fractures has become open reduction and internal fixation [2,3]. Matta reported that the reduction must be precise up to 2 mm for a good clinical outcome, and research is ongoing for safer fixation techniques to avoid unwanted re-displacement [4-7].

Anterior column fractures can be seen as either isolated or accompanied by many types of complex acetabulum fractures. Isolated anterior column fractures are seen in 3%-5% of all acetabular fracture cases [1,4,8]. Regardless of the starting point, whole anterior column fractures pass from the pelvic brim through the quadrilateral surface and disrupt the obturator foramen and are usually treated with suprapectineal plates. Although there have been biomechanical investigations of complex-type acetabular fractures including different subtypes of anterior column fractures and the anterior column in the recent literature, there has been no biomechanical research that investigates the stability provided by different localization of the suprapectineal plate [5,9-12]. This topic is particularly important in clinical practice, as some fracture types necessitate a more lateral location to control the fracture fragments. In cases with an accompanying anterior wall fragment or a fracture of both columns separating the entire pelvic brim from the iliac wing, the surgeon may be challenged by a decision to move laterally apart from or remain close to the brim to ensure stiff and secure fixation.

The main hypothesis is that lateralization of the plate will have no effect on the displacement and stiffness of the fracture fixation on an anterior column fracture according to Letournel’s classification. In this empirical thesis study using bone models, evaluations and comparisons were made of the change in fracture displacement and stiffness, according to the placement of the suprapectineal plate in relation to the pelvic brim.

## Materials and methods

The study was performed after permission was granted by the Sağlık Bilimleri Üniversitesi (SBU) Ankara Training and Research Hospital Council of Ethics for Specialization on September 13, 2017, and the decision of the SBU Academic Council on September 26, 2017. This experiment used 24 pieces of right side, adult, foam cortical shell artificial hemipelvis models (Model 1296-2, Sawbones, Pacific Research Laboratories, Vashon, WA, USA). Three groups of eight artificial hemipelvis models were established in each, as one control group and two fixation groups. The biomechanical tests of the study were performed in the Biomechanics Laboratory of the Union of Chambers and Commodity Exchanges of Turkey (TOBB) University of Economics and Technology Mechanical Engineering Department. Titanium plates and screws were supplied by Normmed Medical Co. (Ankara, Turkey).

Definition of groups and sample sizes

The pelvic models were prepared to measure the effectiveness of two different positions of suprapectineal plate used in the intermediate anterior column fractures. Three study groups were created, which were labeled as M, L, and control groups. Under assumptions of vector dislocation mean (0.03 mm) and standard deviation (0.02), it was concluded from power analysis that eight artificial pelvic models would be needed for each group to reach statistically significant results (b = 0.80, a = 0.005). On the artificial models in group “M,” the suprapectineal plate was placed adjacent to the pelvic brim and was fixed. On the artificial models in group “L,” the suprapectineal plate was placed adjacent to the pelvic brim at the symphysis pubis level, 0.5 cm lateral of the pelvic brim at the fracture line level and 2 cm lateral of the pelvic brim at the sacroiliac joint level. The control group was pelvic models without a fracture.

Creating the fracture models

**Figure 1:**
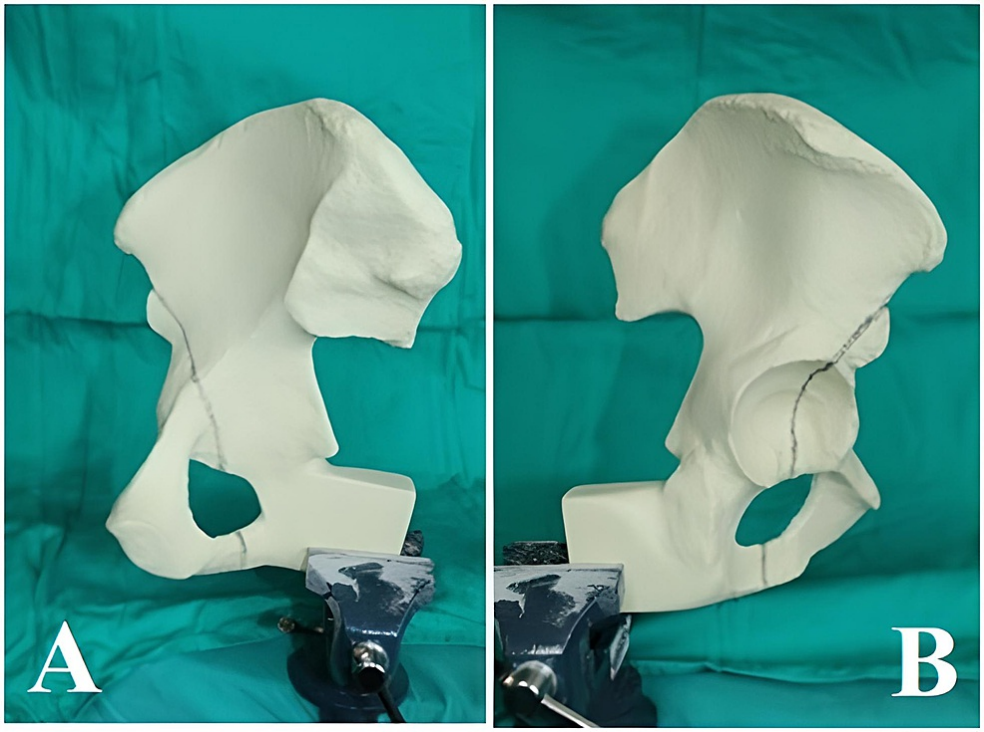
Lateral and medial view of the fracture line (A and B)

Each artificial pelvic model was set up with previously specified fracture lines compatible with intermediate anterior column fracture according to Letournel’s classification. On the lateral view, the fracture line was initiated from the point 15 mm superior to the spina iliaca anterior inferior and was continued as far as 10 mm anterior of the midpoint of the cotyloid fossa and further passed through the midpoint of the acetabulum transverse ligament. The fracture line was maintained in the corresponding ischial ramus (Figure 1A).

**Figure 2:**
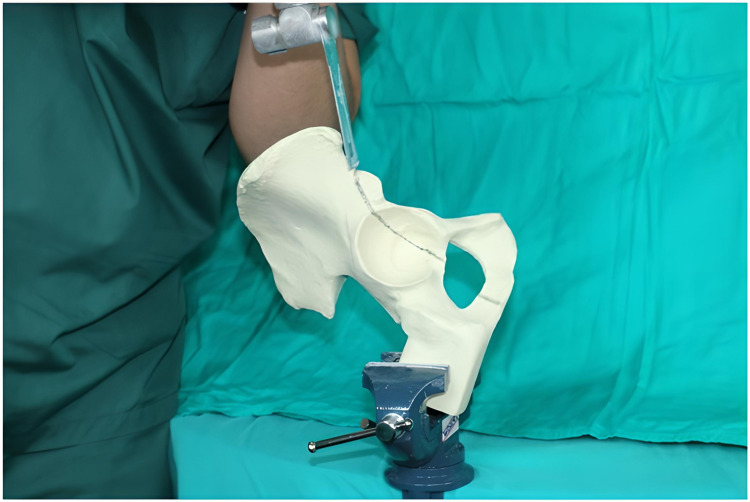
Fracture created using a cutting saw blade on the drawn line

On the medial view, the fracture line was drawn from 15 mm superior of the spina iliaca anterior inferior and was continued from the acetabulum roof to 55 mm anterior from the point of intersection of the pelvic brim and the sacroiliac joint. From this point, it was continued down the quadrilateral surface toward the medial projection of the midpoint of the transverse acetabular ligament (Figure 1B). The fracture was created by using a cutting saw blade on the drawn line (Figure 2).

Regions of interest

**Figure 3:**
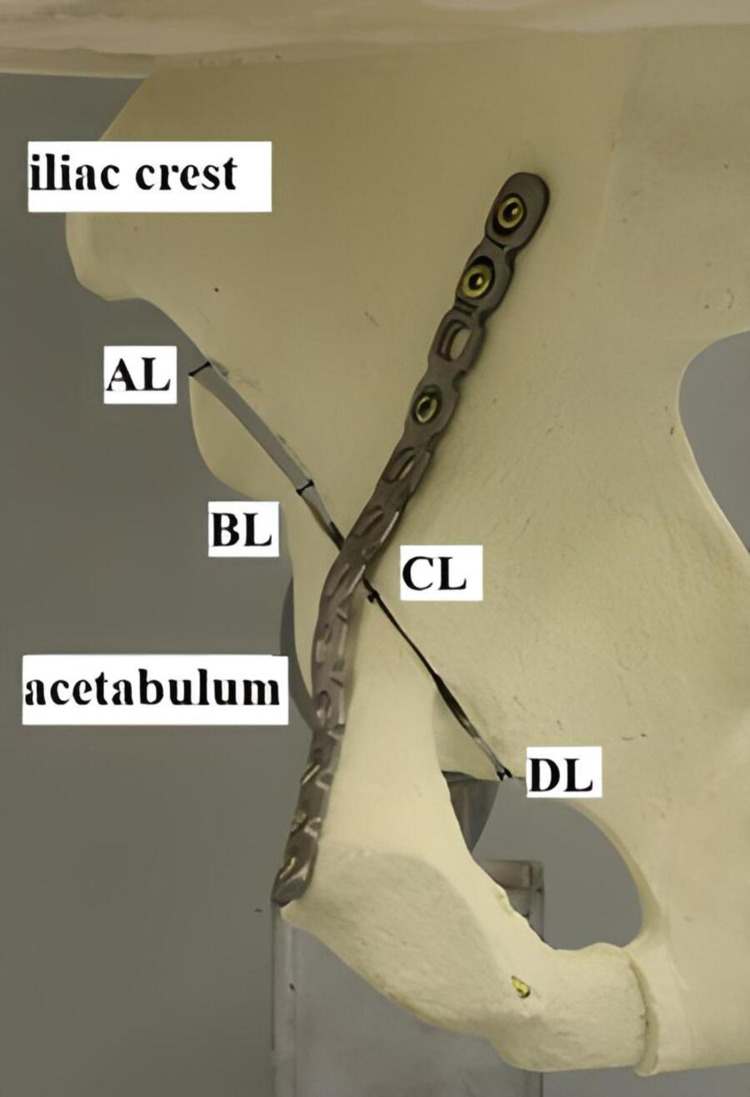
Reference parameters AL, BL, CL, and DL

The AL parameter was determined as the superolateral point of the fracture line, the BL parameter as the superolateral point of the acetabulum joint surface, the CL parameter as the point of contact of the fracture line with the pelvic brim, and the DL parameter as the intersection of the fracture line with the obturator foramen superior border. The reference parameters AL, BL, CL, and DL are shown in Figure 3 for the evaluation of the displacement quantities in the fracture line.

Localization of the plates

**Figure 4:**
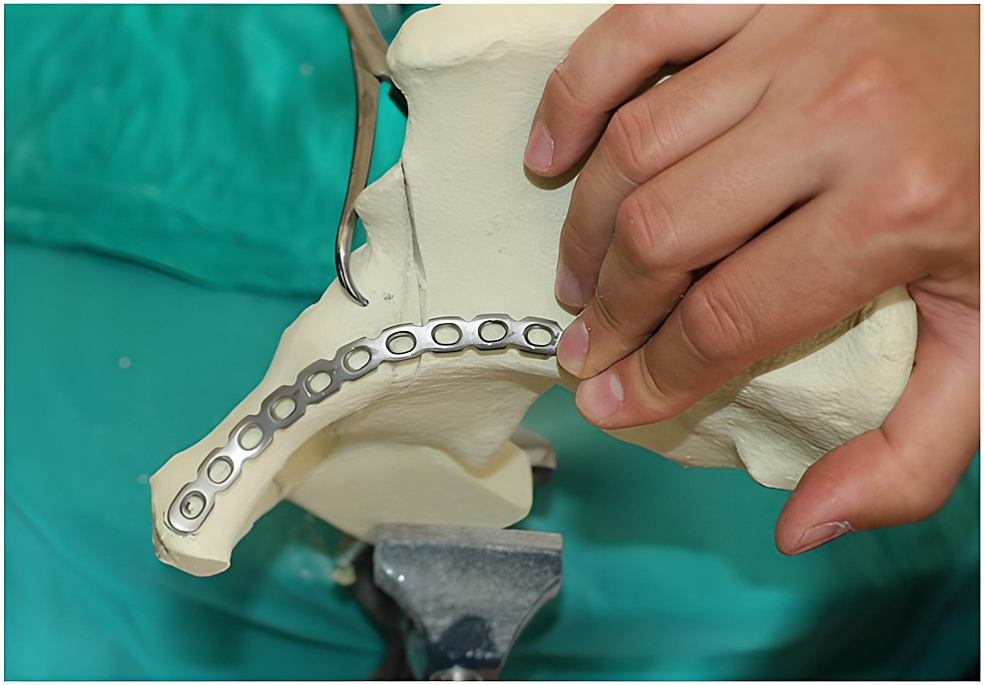
Temporary fixation using reduction clamps

The 3.5-mm, 12-hole curved titanium reconstruction suprapectineal plates to be applied were shaped with flexible templates in accordance with the two different placement points. While the plates in group M were shaped to be positioned adjacent to the pelvic brim up to the sacroiliac joint from the symphysis pubis, the plates in group L were shaped at the level of the symphysis pubis adjacent to the pelvic brim, 0.5 cm lateral to the pelvic brim at the level of the fracture line and 2 cm lateral to the pelvic brim at the level of the sacroiliac joint. The proximal and distal tip points were marked and applied as references for the entire models in each group for consistency. In the next step, the fracture was temporarily fixed using reduction clamps (Figure 4).

**Figure 5:**
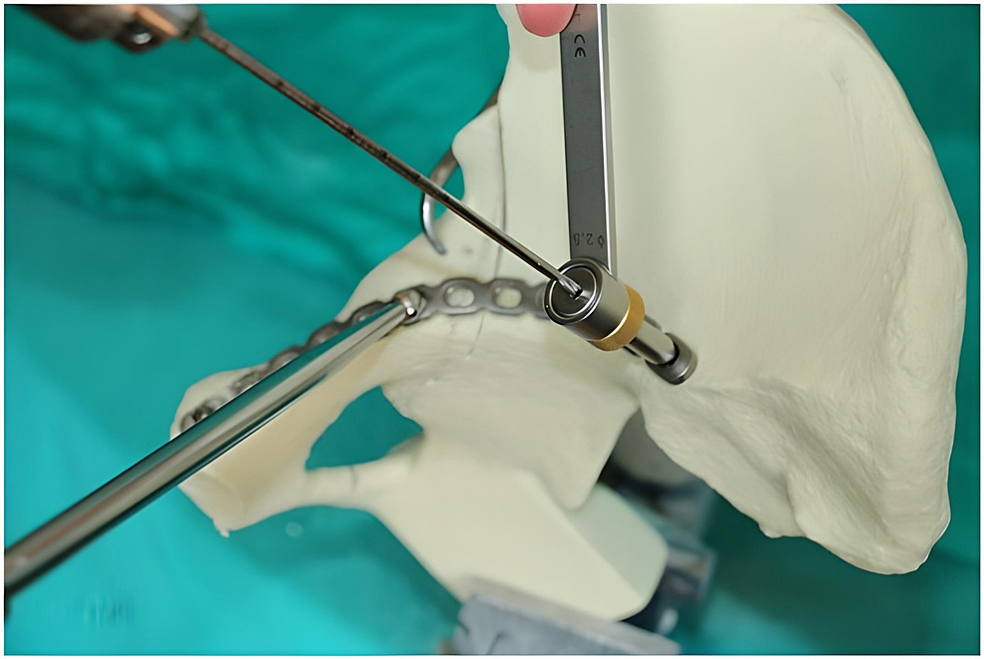
Suprapectineal plate drilled and screwed compressively onto the model

On all the artificial hemipelvis models, the first and last holes of the previously shaped suprapectineal plate were drilled and screwed compressively onto the model (Figure 5).

**Figure 6:**
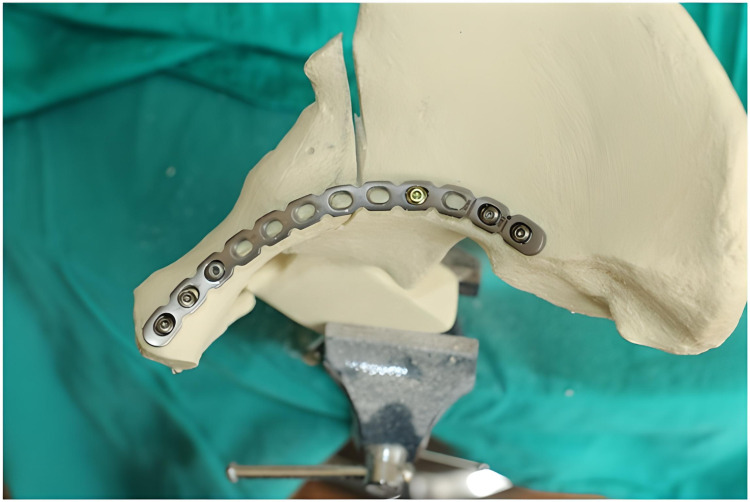
Screw applied through the ninth hole oriented to hold the posterior column

Fixation was achieved with a total of six screws, three on the proximal and distal sides of the fracture (first, second, third, ninth, 11th, and 12th holes). In all the artificial bone models, the screw applied through the ninth hole was oriented to hold the posterior column (Figure 6).

Preparation of the biomechanical test

**Figure 7:**
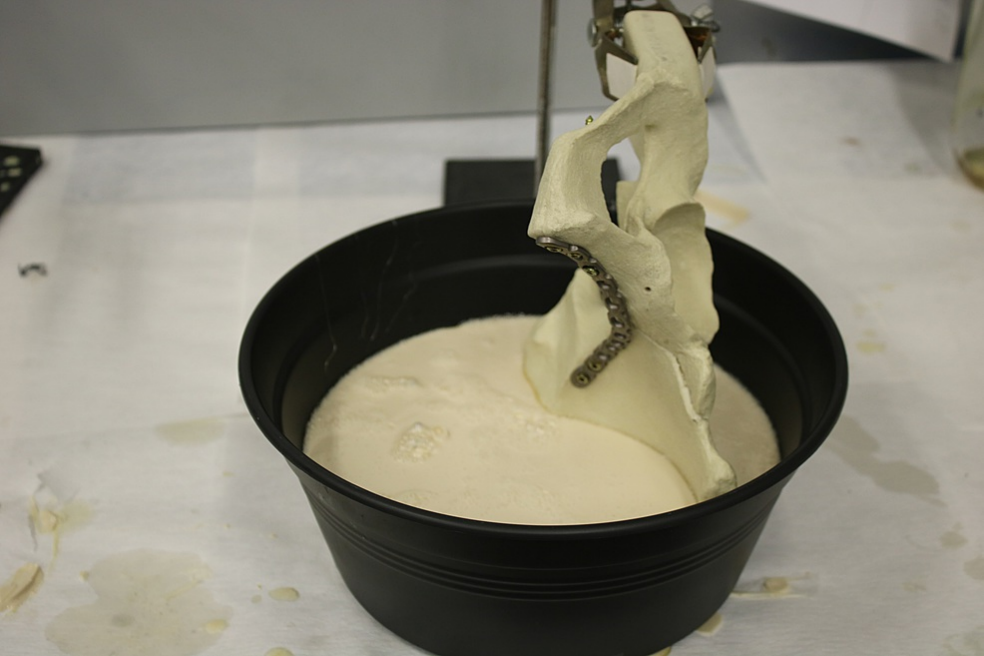
Pelvis fixed at the desired angle

Performing the test under conditions similar to the actual loading conditions is necessary to obtain reliable results. In this respect, the direction of the load applied to the pelvis is critical. The direction of the axial force applied to the hemipelvis models was taken as the angle extending from the lower endplate of the lumbar fifth vertebra to the upper endplate of the first vertebra. There is also the apparatus (1-inch ¼ sleeve) that allows the pelvis to be rigidly connected to the device to be tested in the mold. In order to create a real load center for the device, the rear boundary of the apparatus was placed 2 cm anterior to the posterior boundary of the sacroiliac joint, and the midpoint of the apparatus was placed 4 cm lateral to the anterior border of the sacroiliac joint in the horizontal plane. All the artificial hemipelvis models were stabilized anatomically in the mold with the aid of a special polyurethane material created using isocyanate and polyol (2:3 ratio). Once the mixture material was hardened and ready for use, the pelvis was fixed at the desired angle (Figure 7).

All the hemipelvis models were kept for at least 24 hours in the polyurethane substance before they were taken into the test environment. The stiffness of this polyurethane material, in which the hemipelvis models were held, was adjusted to be higher than that of the hemipelvis models to secure the test results.

**Figure 8:**
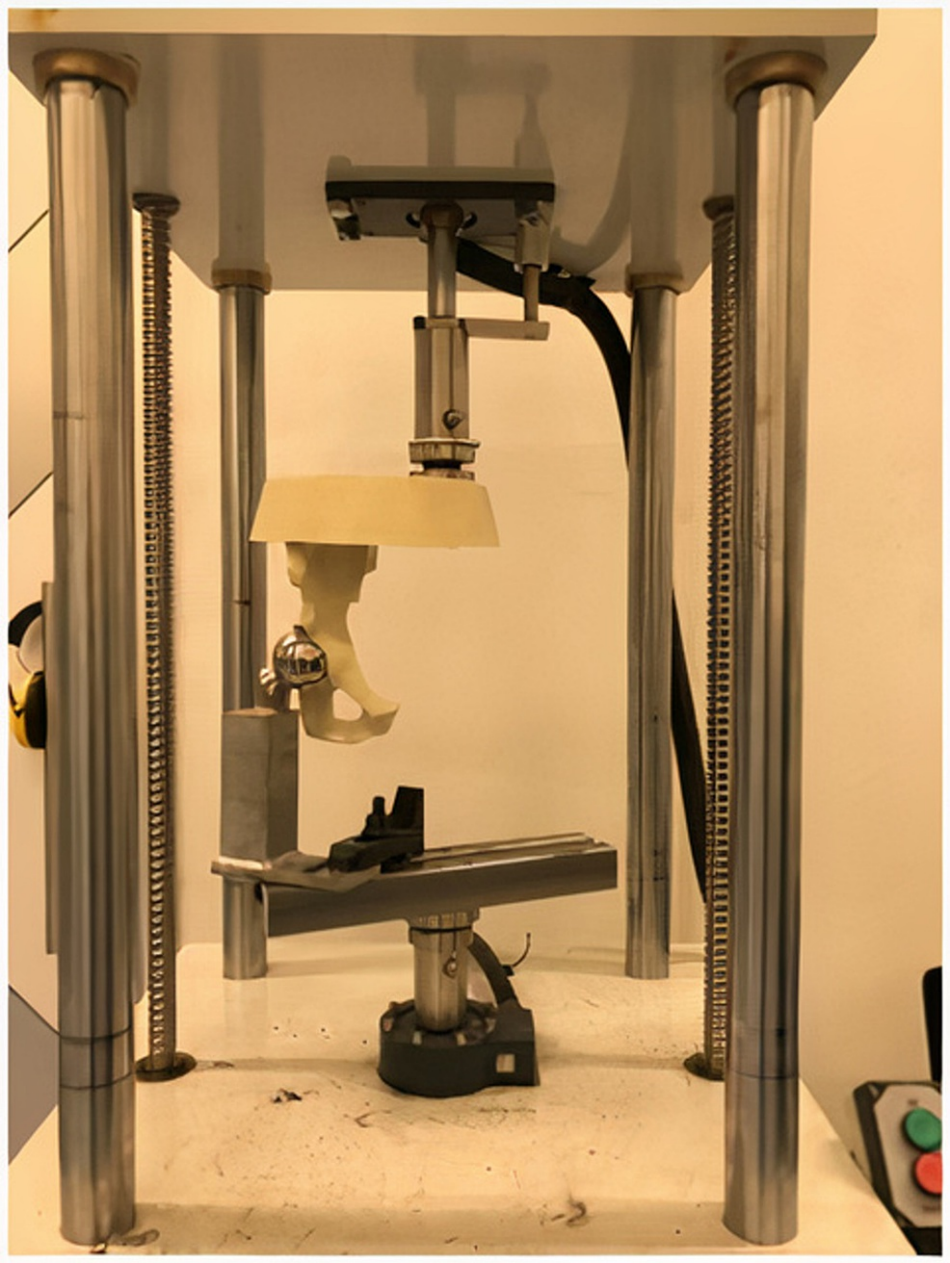
Austin Moore-type prosthesis placed in 15° anteversion into the acetabulum

To evaluate fixation methods under static and dynamic loads, the standing on one leg position was simulated using an Austin Moore-type prosthesis. The Austin Moore-type prosthesis was placed in 15° anteversion into the acetabulum (Figure 8). Dynamic and static tests were conducted on an axial compression device (serial number 2015EMY01, Labiotech, Ankara, Turkey).

Dynamic testing

The anatomically stabilized artificial hemipelvis models were placed on the device in a position to apply force from the midline mimicking the position of the L5 vertebra. Dynamic tests were performed as 1000 cycles at force levels between 50 N and 500 N. During the tests, all the models were photographed with a high-resolution Canon Rebel XSI camera (Canon, Tokyo, Japan), and the AL, BL, CL, and DL values were measured as seen on pre-test and post-test photographs.

Static testing

**Figure 9:**
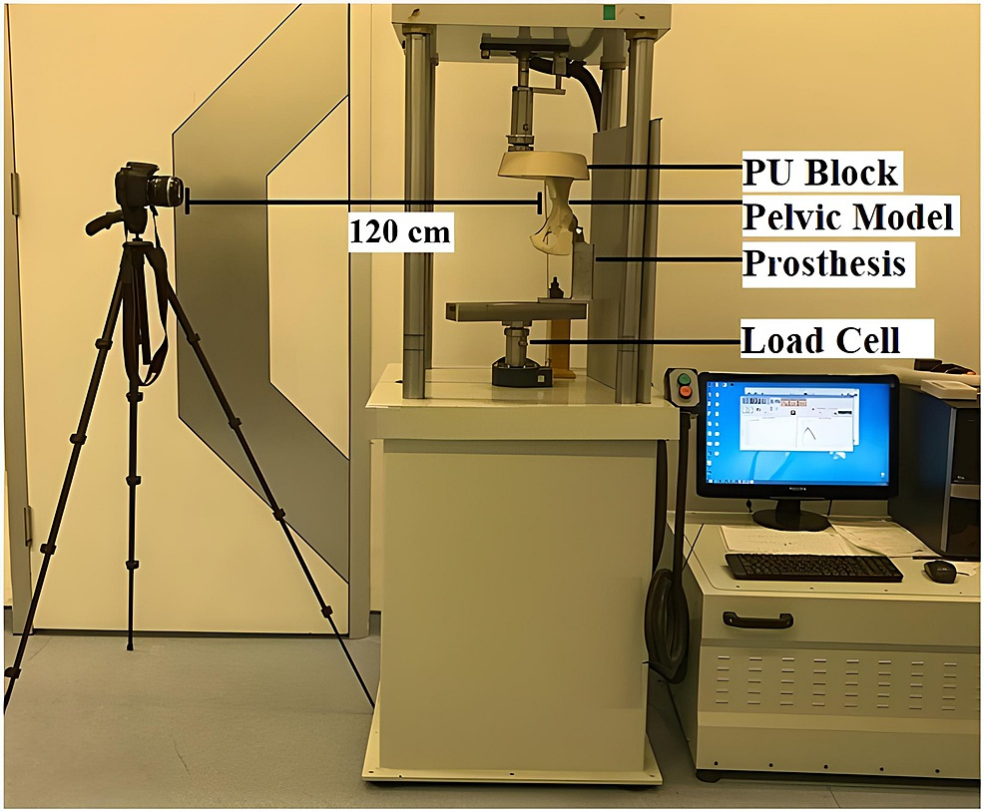
Biomechanical test setup

Following the dynamic tests, each pelvis model was applied a 1.2 kN load at a speed of 2 mm/minute. The maximum load and loading speed were determined by ISO 7206-4. During the tests, a high-resolution camera was placed at a distance of 120 cm, and one picture was taken every second during the loading process. The load versus displacement values were recorded during the test (Figure 9).

Stiffness

The stiffness of the pelvis models was calculated through load-displacement graphics. The inclination of the linear zone in the graph equals stiffness. The values of the control group were used as reference points. The displacement amount in the defined regions of interest (ROI) was measured using Autocad (Autodesk, San Rafael, CA, USA) before and during the loading of the samples. Data analysis was performed using SPSS for Windows version 11.5 (SPSS Inc., Chicago, USA). The nonparametric Mann-Whitney U-test was used for intragroup evaluations. A p-value of <0.05 was considered statistically significant.

## Results

Dynamic loading

The dynamic displacement data after dynamic loading for the AL, BL, CL, and DL parameters are shown below. Table 1 shows the displacement values calculated by subtraction of the values after loading from before loading. For the AL parameter, the median displacement was 0.12 (IQR: 0.058-0.8125) mm in group L and 0.04 (IQR: 0.03-0.065) mm in group M. There was a statistically significant difference between groups L and M in respect of the AL parameter (p = 0.02). There was no difference between the groups in respect of the median values of displacement on BL, CL, and DL points by dynamic loading (Table 1).

**Table 1:** Displacement values and statistical evaluation of reference parameters in dynamic tests * Statistically significant

Reference parameters	Group L		Group M		
	Median	IQR	Median	IQR	p-value
AL	0.12	0.058-0.8125	0.04	0.03-0.065	0.02*
BL	0.115	0.05-0.1150	0.105	0.03-0.1625	0.79
CL	0.13	0.05-0.1300	0.06	0.0225-0.1050	0.09
DL	0.06	0.0125-0.06	0.07	0.0325-0.1375	0.67

Static loading

The displacement values ​​were calculated with the values ​​of the parameters before and after loading. The median and IQR values are given in Table 2. As a result of static loading, no significant difference was found between the two groups in respect of the parameters before and after loading (p > 0.05) (Table 2). The fracture lines in both groups exhibited similar displacement under static load.

**Table 2:** Displacement values and statistical comparison of reference parameters in static tests

Reference parameters	Group L		Group M			
	Median	IQR	Median	IQR	p value	
AL	0.28	0.135-0.40	0.130	0.0250-0.1975	0.093	
BL	0.175	0.093-0.19	0.2150	0.0700-0.4400	0.397	
CL	0.065	0.0150-0.105	0.100	0.0425-0.1500	0.562	
DL	0.120	0.0025-0.210	0.175	0.0625-0.5925	0.188	

The artificial hemipelvis models were grouped as L and M and were tested under dynamic and static loading conditions. Similar displacements were observed for both methods under static loading conditions.

Stiffness

The stiffness of all the groups was calculated with data from load-displacement graphs. The mean stiffness values for the control group, group L, and group M were 474.79 ± 5.84 N/mm, 359.60 ± 43.91 N/mm, and 339.67 ± 56.99 N/mm, respectively. Statistically significantly greater stiffness was determined in the control group compared to groups L and M (p = 0.04 for both). No statistically significant difference was determined between groups L and M in terms of stiffness (p = 0.462). The control group was the most rigid model, and the other two groups showed similar stiffness results (Table 3). Throughout the study, no artificial hemipelvis was broken during either the dynamic or static tests.

**Table 3:** Comparison of stiffness values * Statistically significant

Groups	M versus control	L versus control	M versus L
p-value	0.04*	0.04*	0.462

## Discussion

The suprapectineal region and pelvic brim are the main sites of internal fixation for many types of acetabular fracture and are accessed by anterior approaches. In the treatment of acetabulum fractures, the suprapectineal plate can be placed near the pelvic brim in the medial of the suprapectineal region, or plate application can start from the pubic arm and extend laterally to the iliac crest. The current study was designed to test the effect of lateralization of a curved pelvic brim plate on the stability of an anterior column fracture. The result of the study showed a difference in displacement on the AL point, which is located far from the joint near the spina iliaca anterior inferior. There were no differences between a pelvic brim located plate and a more laterally located plate in articular ROIs in terms of stability. Stiffness was also comparable between the groups. These results may suggest that a curved suprapectineal pelvic brim plate may be located more laterally without serious concerns about the fixation strength, particularly if the fracture pattern necessitates a more lateral location, e.g., some types of both column fractures; these data may help the surgeon in decision-making for any fracture type that includes the anterior column.

Focusing on the displacement of AL in the dynamic test results, despite the fact that the laterally located suprapectineal plate is closer to the AL point, the medially located plate provided better fixation. It was concluded that the better fixation of the medially located plate was mainly due to the proximal screws. On laterally located plates, the proximal screws are delivered to the shorter and lower density bones of the iliac crest, and even the posterior column screw passes from the posterior wall to reach the posterior column. However, on medially located suprapectineal plates, the proximal screws are fixed to thicker, stronger, and longer bone corridors at the pelvic brim. Current literature specifies that the conventional plate and periarticular lag screws placed in the posterior column are the best fixation methods for anterior column fixation [9,13,14]. Another reason might be the angle between the fracture line and the plate [3]. More perpendicular installation of medial suprapectineal plates to the fracture line may have provided an effective solution for rigid fixation. In this study, the mean angles of the plates and the fracture line were 74.5° for medially located plates and 58.6° for laterally located plates.

Since the ilioinguinal and anterior intrapelvic approaches were defined by Letournel and Hirvensalo respectively, various configurations of suprapectineal plate have been used [15-17]. In the experience of the senior author of this study, the localization of a long 12 or 14 suprapectineal plate should be decided according to the following features: A) fracture pattern (e.g., both column fractures often need more laterally localized plates, as the superomedial part of the iliac wing remains as an intact bridge bound to the axial skeleton, where mostly the pelvic brim is damaged and separated); B) the presence of an anterior wall fracture, which is often problematic and necessitates a relatively laterally located suprapectineal plate; C) owing to the general condition of the patient or his/her own experience, the surgeon might wish to locate the plate a few centimeters more laterally to the pelvic brim and use AIPa with a lateral window with a minimally invasive approach, avoiding posterior dissection on the pelvic brim [18].

As a brief summary of the study model, the fracture pattern was selected as an intermediate subtype to encompass all subtypes of anterior column fractures. Biomechanically, the load on the hips is transmitted from two sources: body weight and abductor moment. In the position of standing still on one foot, the two forces remain in balance. The joint reactive force is the sum of the mechanical loads to the hip. The joint reactive force is 2.5-2.8 times the body weight when walking and 0.1-0.5 times the body weight in the swing phase of the walk [19-21]. According to a study on walking analysis, the force applied to the acetabulum at different periods of walking has been found to be between 0.11 and 3.12 times the body weight [22]. In the literature that has compared the relative stiffness and strength of different fracture fixations, there is heterogeneity in the number of experimental loading and cycles [9,10,13,23-25]. In this study, the hemipelvis models were subjected to 1000 cycles of between 50 and 500 N forces for dynamic tests, and a 2 mm/minute speed of 1.2 kN load was applied for static tests, as described by Tanoğlu et al. [5]. When the stiffness values of each group were compared, there was no significant difference between groups L and M. However, both groups provided higher stiffness than the control group. Throughout the current study, no fracture developed in any of the artificial hemipelvis models during either the dynamic or static tests. This proves that both installation methods provide enough stiffness for the suprapectineal plate to sustain a 1.2 kN load.

The limitations of this study are primarily the disadvantages of artificial foam pelvis models, which include the absence of muscles, tendons, and ligaments, the effects of physiological stress during tests, and the inability to evaluate resistance in osteoporosis [26]. However, they are often preferred over cadaver studies, which have their own limitations such as supply issues, danger of infection, inability to standardize anatomical structures, and ethical problems [9,23,26]. The most important advantage of artificial foam pelvis models is that they are closer to normal bone quality in both macroscopic and microscopic aspects.

## Conclusions

In conclusion, both medial and lateral installation of a suprapectineal plate does not affect the fixation stability at the articular part in anterior column fractures under static or dynamic loading. These data might supply some freedom to the operating surgeon in intraoperative planning for these fractures. A lateralized suprapectineal curved plate may have some implications at the uppermost iliac part of the fracture in the case of early weight-bearing, while the articular surface remains stable.
